# Multimodal intraoperative neuromonitoring during total hip arthroplasty in severe hip deformities: how do we do it and when do the alerts occur?

**DOI:** 10.1007/s00402-025-06060-y

**Published:** 2025-09-29

**Authors:** Tim T. C. R. de Mees, Vincent J. J. F. Busch, Juerd Wijntjes, Petra J. C. Heesterbeek, Rian Grutters, Ilona Racz, Leon van Loon, Marc W. Nijhof

**Affiliations:** 1 Department of Orthopaedic Surgery, Sint Maartenskliniek, Nijmegen, Netherlands; 2 Department of Orthopaedic, Radboud University Medical Center Nijmegen, Geert Grooteplein Zuid 10, 6525 GA Nijmegen, Netherlands

**Keywords:** Intraoperative neuromonitoring, Neuromonitoring, IONM, Multimodal IONM, Total Hip Arthroplasty, DDH, Nerve injury, Hip deformity, Motor evoked potentials, Leg lengthening

## Abstract

**Introduction:**

Total Hip Arthroplasty (THA) with leg lengthening can offer great outcome in patients with severe hip deformities but comes with higher risk of intra-operative nerve injury. The aim was to investigate the relationship between surgical steps and multimodal intraoperative neuromonitoring (IONM) events during THA in severe hip deformities.

**Methods:**

This cohort study of 27 cases with symptomatic severe hip deformities, such as Crowe 3 and 4 developmental dysplasia of the hip (DDH). All cases received THA with IONM between September 2019 and December 2023. The goal of surgery in all cases was to reestablish the original center of rotation. Monitored modalities were Transcranial Motor Evoked Potentials (TcMEPs), Somatosensory Evoked Potentials (SSEPs) and free-running Electromyography (EMG). Alarm criteria were a TcMEP amplitude loss of 80%, neurotonic discharges on the free-running EMG and/or significant SSEP amplitude loss. Follow-up took place during hospital stay and at the follow-up visit 8 weeks postoperatively.

**Results:**

In 16 of 27 cases we found IONM signal alerts that correlated with several surgical events (in total 22 events): surgical exposure/hip dislocation (12 events), acetabular reconstruction/cementing (4 events) and trial reduction (6 events). Actions taken were removal or replacement of instruments, neutralization of the leg, screw replacement of the massive autograft (2 events) and extensive subtrochanteric osteotomy (4 events). In 7 cases the IONM alerts concerned solely sciatic innervated muscles, in 2 cases solely femoral and in 7 cases both sciatic and femoral innervated muscles. In 14 cases normalization of IONM signals occurred spontaneously or after corrective maneuvers, in 2 cases no normalization occurred during surgery. No case showed a postoperative neurological deficit at follow up.

**Conclusion:**

Multimodal IONM in THA for severe hip deformities revealed numerous specific surgical steps leading to IONM signal alerts. In a majority of cases adjustment of the surgical procedure could normalize the IONM signals. None of our patients experienced a postoperative neurological deficit.

## Introduction

Primary Total Hip Arthroplasty (THA) with lower-extremity lengthening can offer excellent outcome in patients with severe hip deformities, such as severe developmental dysplasia of the hip (DDH) [1–8]. However, the distorted anatomy and sometimes considerable leg length discrepancy increase the risk of intraoperative nerve injury substantially [3, 6, 9–14]. Nerve injury during THA can result from traction, compression, ischemia or transection and mostly involves the sciatic and / or femoral nerve. Nerve injury can exist of complete or incomplete palsies or dysesthesia and recovery can take up to 18 months or does not occur at all in case of severe axonal loss [11].

A retrospective analysis in our tertiary orthopedic center of patients who received a THA for DDH between 2012 and 2018 showed that the rate of postoperative nerve injury was 11% in 18 patients with severe DDH rated as Crowe 4 or Hartofilakidis C1/C2 versus 2% in the total DDH group of 308 patients. Similar to our findings, literature shows that nerve palsies after THA in severe DDH had a 4 times higher odds of perioperative nerve injury and rates ranged from 1 to 13% [1, 3, 4, 6, 8–13, 15, 16]. Aiming to reduce the incidence of nerve injury we started using multimodal intraoperative neuromonitoring (IONM) during THA for severely deformed hips (such as in severe DDH) in 2019.

IONM has shown to be beneficial in performing safe and adequate lower-extremity lengthening during specific cases of THA [12, 13, 16–20]. Neuromonitoring during surgery provides instant feedback on (potential) neural function loss, allowing for subsequent corrective maneuvers [12, 13, 19–21]. A lot is yet unknown about which specific surgical steps are threatening for nerve injury, which specific IONM alerts subsequently occur and what the potential corrective effect of surgical responses is to these alerts. Although multimodal (as opposed to only a single type of) IONM might increase efficiency, this technique has not been studied widely in THA [18, 19, 21, 22]. In this case series we report about the feasibility of the use of multimodal IONM during primary THA for severe hip deformities. The aim was to analyze multimodal IONM alerts: Which specific surgical steps (maneuvers or instrumentation) lead to these alerts? To what extent did subsequent surgical corrective maneuvers lead to recovery of the alerts? Furthermore, the association between intraoperative events and possible postoperative neurological deficits was studied.

## Methods

### Patients

This cohort study describes all patients who received THA with multimodal IONM between September 2019 and December 2023 (*n* = 27). Patients were considered eligible for IONM if they had symptomatic severe/high-riding DDH (Crowe 3/4 or Hartofilakidis B2/C1/C2) or a highly distorted hip anatomy due to other pathology or an apparent preoperative leg length difference of ≥ 2 cm. When patients gave permission for the use of IONM, they were evaluated by the IONM-supervisor/neurologist (JW) preoperatively to verify appropriate IONM indication. After informed consent by letter and/or verbally, 1 patient declined participation in this study and was not included.

### Surgical technique

**Fig. 1:**
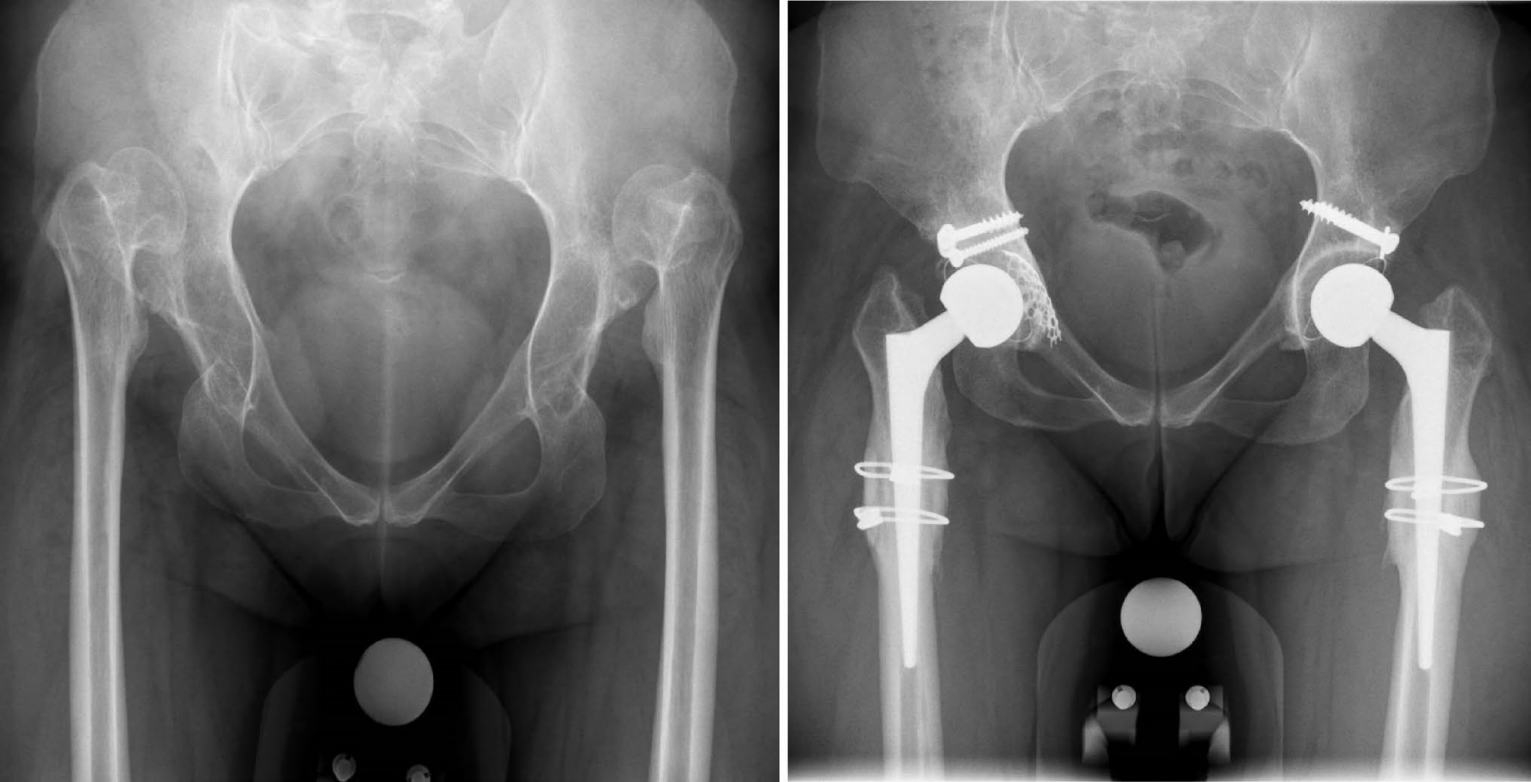
Pre- and postoperative X-ray of a patient with Crowe 4 DDH. The postoperative X-ray shows restoration of the original center of rotation (COR). In this patient bilateral massive autografts were placed and bilateral subtrochanteric osteotomy was performed

The goal of surgery in all cases was to reestablish the original center of rotation by reconstructing the original acetabulum resulting in possible lengthening of the limb (Fig. 1). A posterolateral approach was used in all cases by two experienced hip arthroplasty surgeons (MN and VB). Defects of the anatomical acetabular roof of over 20% were corrected with a bulk femoral head autograft to fill the superolateral defect [6, 23]. The majority of cases received a cemented polyethylene cup (M.E. Müller Durasul Low Profile, Zimmer Biomet, USA). A transverse subtrochanteric osteotomy was performed if substantial limb lengthening was foreseen (estimated above 2 centimeters), if there would be an expected unacceptable leg length difference postoperatively or if the surgeon expected a high risk of nerve injury during surgery [5, 7, 15, 24, 25]. Depending on the estimated degree of limb lengthening, a subtrochanteric fragment between 2 and 4 centimeters in length was resected. More subtrochanteric resection was performed when muscular tensions remained too high or when IONM signals decreased significantly during trial reduction (see the description below). After trial reduction and placement of the definite femoral stem, the femoral osteotomy fragment was split, placed around the osteotomy cut and fixated with two cables. The majority of cases received a short cone tapered uncemented stem (Wagner Cone, Zimmer Biomet, USA) [5].

### IONM technique

All patients received total intravenous anesthesia. Inhalational anesthetics such as sevoflurane or desflurane can interfere with IONM signals and were therefore avoided [26]. In all cases, IONM was performed using a NIM-Eclipse E4 neuromonitoring system (Medtronic, Minneapolis, USA).

**Fig. 2:**
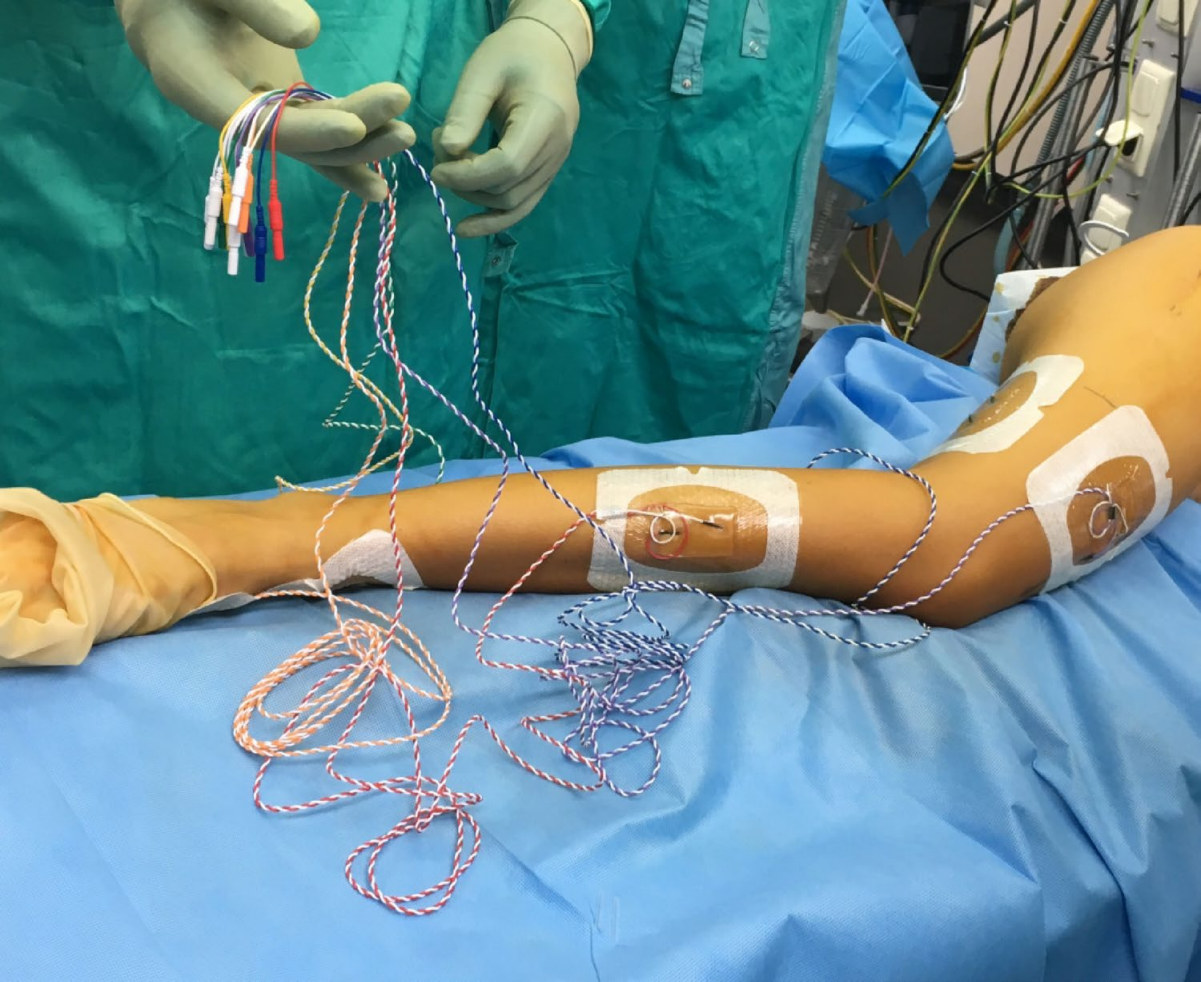
Sterile placement of the electrodes

Monitored modalities were Transcranial Motor Evoked Potentials (TcMEPs), Somatosensory Evoked Potentials (SSEPs) and free-running Electromyography (EMG). To stimulate the TcMEPs, corkscrew electrodes were placed on the skull. Needle electrodes were placed bilaterally in the biceps femoris, gastrocnemius, abductor hallucis and tibial anterior muscle to monitor sciatic nerve function, and in the rectus femoris muscle to monitor femoral nerve function. As a control muscle the m. extensor carpi radialis was used. To monitor the SSEPs, the tibialis posterior nerve was stimulated at the level of the ankle and recorded from the sensory cortex (CpZ-CP3/4). A free running EMG monitored spontaneous muscle activity of the same muscles that were monitored with the TcMEPs. All electrodes on the operated leg were placed sterile (Fig. 2).

Baseline TcMEP and SSEP signals were recorded before the start of the surgery to provide a reliable reference for comparison with subsequent intraoperative measurements.

TcMEPs were elicited every 5 min and upon surgical demand. Alarm criterium was a TcMEP amplitude loss of 80% compared with baseline or a relative or asymmetrical change in the amplitude of the TcMEP [27]. In SSEPs, alarm criteria was a significant visual apparent amplitude loss compared to pre-change values [28]. Furthermore, the surgeon was informed when sustained neurotonic discharges were seen on the free-running EMG.

### Clinical follow-up

Screening for signs of possible neurological complications took place during hospital stay and at the first follow-up visit that was scheduled 8 weeks postoperatively. Signs of nerve injury included motor weakness in muscles and / or sensory deficits in the skin area innervated by the sciatic and/or femoral nerve. When patients did not report any nerve related complaints or did not have any loss of sensory or motor function during hospital stay or at the first follow up, they were considered to have no nerve injury.

## Results

### Demographics

In total, 27 cases (25 patients; 2 bilateral THAs) were included. The mean age at surgery was 34.8 years and the mean BMI at surgery was 25.6. Twenty-one of 27 cases had hip deformities due to DDH (mostly graded as Crowe 3 or 4 or Hartofilakidis B2, C1 or C2). The other 5 cases had hip deformities secondary to other (childhood) diseases like Perthes disease or septic arthritis (Table 1). The majority of patients received a femoral head bulk autograft and/or underwent a femoral shortening osteotomy (Table 2).

**Table 1:** Demographics and (classification of) diagnosis of the patients

Study number	Age at surgery	Gender	Height (m)	Weight (kg)	BMI	Side (of surgery)	Primary diagnosis	Crowe	Hartofilakidis	Previous ipsilateral hip surgery
1	20	F	1.58	48	19.2	R	DDH	4	C1	Yes
2	31	M	1.84	68	20.1	L	DDH	3	B2	Yes
3*	39	F	1.58	70	28.0	L	DDH	4	C2	No
4	23	M	1.78	120	37.9	R	Septic arthritis	4	C2	No
5	30	F	1.72	82	27.7	R	DDH	3	B2	Yes
6*	40	F	1.58	73	29.2	R	DDH	4	C2	No
7**	56	F	1.65	53	19.5	L	DDH	4	C2	Yes
8	27	M	1.63	83	31.2	L	DDH	4	C2	No
9	53	M	1.56	61	25.1	L	DDH	4	C1	No
10	55	F	1.75	95	31.0	R	DDH	2	B2	No
11**	57	F	1.66	55	20.0	R	DDH	4	C2	No
12	16	M	1.66	76	27.6	L	DDH	4	C1	Yes
13	17	M	1.72	59	19.9	R	DDH	1	B1	Yes
14	35	F	1.79	117	36.5	R	DDH	3	B2	No
15	37	F	1.58	52	20.8	L	DDH	2	B1	Yes
16	23	F	1.5	42	18.7	R	Pseudoreumatoid dysplasia	1	B1	Yes
17	33	M	1.8	81	25.0	R	Osteomyelitis	1	A	No
18	28	F	1.67	59	21.2	R	Perthes	3	B2	No
19	20	F	1.73	58	19.4	R	DDH	4	C1	Yes
20	28	F	1.56	58	23.8	L	DDH	3	B2	Yes
21	59	M	1.77	92	29.4	R	DDH	2	B1	Yes
22	57	F	1.66	61	22.1	L	DDH	1	B1	No
23	34	M	1.62	67	25.5	R	DDH (secondary to CP)	3	B2	No
24	38	M	1.71	69	23.6	L	DDH (secondary to CP)	3	B2	Yes
25	35	F	1.71	64	21.9	R	SCFE	1	B1	Yes
26	29	F	1.3	55	32.5	R	Multiple epiphyseal dysplasia	2	B1	No
27	19	F	1.63	83	31.2	L	DDH	3	B2	No

*F* female, *M* male, *BMI* Body Mass Index, *L* left, *R* right, *DDH* Developmental Dysplasia of the Hip, *CP* Cerebral Palsy

*: bilateral cases of the same patient.**: bilateral cases of the same patient.

**Table 2:** Surgery and distalization/lengthening data

Study number	Duration of surgery (min)	Massive acetabular autograft	Subtrocanteric osteotomy	Greater/lesser trochanter distalization (mm)**	Osteotomy length (mm)***	Leg lengthening (mm)****
1	93	No	No	26	-	26
2	115	Yes	No	23	-	23
3	202	Yes	Yes	61	35	26
4	285	Yes	Yes	47	30	17
5	132	Yes	No	22	-	22
6	203	Yes	Yes	59	38	21
7	154	No	Yes	67	30	37
8	175	No	Yes	72	31	41
9	195	No	Yes	63	41	22
10	96	Yes	No	8	-	8
11	162	Yes	Yes	63	30	33
12	190	Yes	Yes	47	27	20
13	117	Yes	No	21	-	21
14	110	Yes	No	18	-	18
15	135	Yes	No	19	-	19
16	165	Yes	Yes	45	36	9
17	181	No	Yes	45	27	18
18	170	Yes	Yes	38	30	8
19	136	Yes	No	13	-	13
20	94	No	No	22	-	22
21	129	Yes	No	34	-	34
22	96	Yes	No	0	-	13
23	160	No	Yes	28	25	3
24	393 (part of SEMLS*)	Yes	Yes	30	25	15
25	107	Yes	No	5	-	5
26	107	Yes	No	29	-	29
27	171	Yes	Yes	0	30	-2

*: Single-Event Multi-Level Surgery **: Trochanter distalization was measured on (calibrated pre- and postoperative X-rays. ***: Osteotomy length was the measured length of the subtrochanteric segment removed during surgery. ****: Leg lengthening was measured by subtracting osteotomy length of trochanter distalization

### IONM alerts

In 16 of 27 cases one (*n* = 5) or more (*n* = 11) IONM signal alerts occurred. Alerts occurred in TcMEPs alone in 12 cases, and in both TcMEPs and SSEPs in 4 cases. The IONM signal alerts were correlating with the following surgical events and subsequent surgical responses (Table 3):

1. During/after surgical exposure and hip dislocation (12 events): in all cases the signal alert was followed by a temporal removal or replacement of instruments.
2. During/after acetabular reconstruction (4 events): in all cases the signal alert was followed by a temporal removal or replacement of instruments with or without neutralization of the leg and in 2 cases one of the cancellous screws to fixate the massive graft was replaced and repositioned.
3. During trial reduction after cup placement (and subsequent distalization of the center of rotation) (6 events): this led to an additional shortening osteotomy in 4 cases following the initial subtrochanteric osteotomy. In 1 other case the trial stem was placed deeper and the IONM signals were heightened. In the other case the signals normalized after replacement of instruments and flexion of the leg.

**Table 3:** TcMEP alerts, surgical correlation and actions and postoperative nerve injury

Study number	IONM sciatic alert *	IONM femoral alert **	Number of alerts	Duration longest alert (min)	Surgical correlation	Surgical action	Postoperative nerve injury
1	No	No	0	NA	NA	NA	No
2	No	No	0	NA	NA	NA	No
3	Yes	Yes	2	45	Surgical exposure and hip dislocation	Temporal removal of instruments, neutralization of the leg	No
4	Yes	Yes	2		Reposition after distalization of COR	More subtrochanteric osteotomy (limited lengthening)	No
5	No	No	0	NA	NA	NA	No
6	Yes	Yes	2	6	1. surgical exposure and hip dislocation 2. reposition after distalization of COR	1. temporal removal of instruments and neutralization of the leg 2. limited lengthening	No
7	No	Yes	1	1	Hip dislocation	Temporal removal of instruments	No
8	Yes	No	2	8	Reposition after distalization of COR (twice)	More subtrochanteric osteotomy (limited lengthening) and deeper placement of stem	No
9	No	No	0	NA	NA	NA	No
10	Yes	No	2	3	Hip dislocation	Temporal repositioning of the hip	No
11	Yes	Yes	3	15 (no normalization at end of surgery)	1. hip dislocation 2. reposition after distalization of COR	1. temporal removal of instruments 2. more subtrochanteric osteotomy (limited lengthening)	No
12	Yes	No	2		1. epidural placement 2. surgical exposure/ cementing	Temporal removal of instruments	No
13	Yes	No	1		Surgical exposure/ cementing	Temporal removal of instruments	No
14	No	No	0	NA	NA	NA	No
15	Yes	No	6	12	Surgical exposure and hip dislocation	Temporal removal of instruments	No
16	Yes	No	1	4	1. surgical exposure 2. construction of acetabulum	1. temporal removal of instruments 2. replacement OSM acetabulum	No
17	No	No	0	NA	NA	NA	No
18	Yes	Yes	1	2	1. surgical exposure and hip dislocation 2. reposition after distalization of COR	Temporal removal of instruments and neutralization of the leg	No
19	Yes	Yes	1	3	Reposition after distalization of COR	1. temporal removal of instruments and neutralization of the leg 2. heigthening of signals 3. deeper placement of stem	No
20	No	No	0	NA	NA	NA	No
21	Yes	Yes	4	47 (no normalization at end of surgery)	No surgical correlation, decreased signals during entire operation	Temporal removal of instruments	No
22	No	No	0	NA	NA	NA	No
23	No	No	0	NA	NA	NA	No
24	No	Yes	2	13	Surgical exposure	Temporal removal of instruments	No
25	No	No	0	NA	NA	NA	No
26	Yes	No	2	7	1. surgical exposure 2. construction of acetabulum	1. temporal removal of instruments 2. replacement OSM acetabulum	No
27	No	No	0	NA	NA	NA	No

*: < 20% of baseline TcMEP amplitude of sciatic innervated muscle. **: < 20% of baseline TcMEP amplitude of femoral innervated muscle. NA: not applicable

In seven cases the IONM alerts concerned only sciatic innervated muscles, in seven cases both sciatic and femoral innervated muscle IONM alerts were seen and in two cases the IONM alerts only concerned the femoral innervated muscles. The duration of IONM alerts ranged between 1 and 47 min. In 14 of 16 cases normalization of IONM signals occurred spontaneously or after corrective maneuvers during surgery. In 1 case (case 11, Table 3) the TcMEP amplitude of the m. quadriceps (femoral nerve) dropped after reposition and remained below the alarm criterium. Increasing the subtrochanteric osteotomy did not result in improvement of the TcMEP amplitude. After collective consideration of surgeons, neurologist and IONM team, this ongoing alert was accepted as there was no perception of excessive leg lengthening. The amplitude remained below the alarm criterium (10–21%) until the end of surgery. In 1 other case (case 21, Table 3), the TcMEP amplitudes of the right m. quadriceps, m. tibialis anterior, m. gastrocnemius, m. adductor hallucis and also the left m. quadriceps alerted since the start of surgery. No surgical correlation was found and there were no changes in the anesthesia administered to the patient. After collective consideration of surgeons, neurologist and IONM team, these ongoing alerts were accepted. The signals of the m. quadriceps and m. gastrocnemius remained under alarm criteria until the end of surgery.

### Clinical follow-up

None of the 27 cases showed a postoperative neurological deficit of the sciatic or femoral nerve, either directly postoperatively or at follow-up at 8 weeks postoperatively.

## Discussion

Multimodal IONM during THA for severe hip deformities can provide surgeons with accurate feedback and alerts about relevant nerves and the impact of surgical actions during surgery. In the majority of cases in present series, the intraoperative responsive actions of surgeons following IONM alerts were subsequently followed by restored IONM signals to baseline. This fact, and the absence of postoperative neurological complications, suggested IONM to be a contributing factor for a safe THA procedure and accompanying leg lengthening in severe hip deformities.

The risk of neurological complications after THA in severe hip deformities may be substantial [1, 3, 6, 8–12]. However, some studies showed lower rates of nerve injury after THA in such cases, questioning the need for IONM monitoring [4, 25, 29]. Eggli et al. showed there was no correlation between the degree of leg lengthening and nerve injury [30]. However, Kong et al. and Rasi et al. showed a trend in reduction of nerve injury by usage of IONM. They also observed a significantly higher degree of leg lengthening (and subsequent correction of leg length difference) in patients operated with IONM [12, 16]. Furthermore, the studies of Murena et al., Taheriazam et al. and Turan et al., all agree on the importance of IONM in identifying and acting upon potential nerve injury during surgery, in line with our results [18–20].

Due to the methodological setup of our study, we cannot provide evidence whether the surgical responses were actually necessary to normalize IONM signals and avoid symptomatic nerve injury. In two cases a false positive persistent IONM alert did not lead to symptomatic nerve injury. This was also described in a recent study by Taheriazam et al. [19]. In both cases in our study the m. quadriceps muscle was involved. We deliberately selected the m. quadriceps to monitor femoral nerve function. However, its relatively sparse corticospinal innervation, compared to, for example, the m. abductor hallucis, makes it more prone to false positive measurements, which may have influenced our findings [31]. A larger study is needed to confirm the actual contribution of multimodal IONM in prevention of neurological complications after THA for severe hip deformities.

Of note, the threshold of an 80% decrease in TcMEP amplitude that we used as a warning criterion is based on TcMEP measurements during scoliosis surgery and aims to strike a desirable balance between preventing unwanted deficits and avoiding false-positive readings [32]. The false-positive rate (2/27) in our study is roughly comparable to the reported false positive rate in the quadriceps muscles in a recent study involving patients undergoing low lumbar spinal surgery. In this study by Allison et al., TcMEPs were compared with transabdominally stimulated MEPs (TaMEP), the latter showing significantly fewer false positives; possibly because this method is less affected by general anesthesia [33]. This technique can also be applied in hip surgery [34]. Further research into the use of TaMEP monitoring in hip surgery therefore seems both desirable and promising.

The extra costs (materials, staff) and preparation time of multimodal IONM (placing of electrodes, measurement validation/controls, between 15 and 30 min per case) and the mandatory general anesthesia are limitations of (the use of) multimodal IONM. Focusing on these potential drawbacks, a topic of interest for further research would be to compare the current multimodal IONM technique with alternative IONM techniques such as TaMEPs in which spinal anesthesia can be used [34]. Since IONM has not been widely used in THA yet, larger clinical trials and health technology assessments could provide important further insights to determine its benefits, to further validate measurement thresholds and to optimize the implementation of IONM in complex THA cases [18].

## Conclusion

Multimodal IONM in THA for severe hip deformities revealed numerous specific surgical maneuvers (including, but not restricted to actual leg lengthening itself) leading to IONM signal alerts. In a majority of cases adjustment of the surgical procedure was followed by normalization of the IONM signals. None of our patients experienced a postoperative neurological deficit.

## Data Availability

Data is available upon reasonable request at the corresponding author.

## References

[1] Eskelinen A, Helenius I, Remes V et al (2006) Cementless total hip arthroplasty in patients with high congenital hip dislocation. J Bone Joint Surg Am 88:80–91. 10.2106/JBJS.E.00037 16391252 10.2106/JBJS.E.00037

[2] Greber EM, Pelt CE, Gililland JM et al (2017) Challenges in total hip arthroplasty in the setting of developmental dysplasia of the hip. J Arthroplasty 32:S38–S44. 10.1016/j.arth.2017.02.024 28291651 10.1016/j.arth.2017.02.024

[3] Mu W, Yang D, Xu B et al (2016) Midterm outcome of cementless total hip arthroplasty in Crowe IV-Hartofilakidis type III developmental dysplasia of the hip. J Arthroplasty 31(20151026):668–675. 10.1016/j.arth.2015.10.011 26643734 10.1016/j.arth.2015.10.011

[4] Wang D, Li LL, Wang HY et al (2017) Long-Term results of cementless total hip arthroplasty with subtrochanteric shortening osteotomy in Crowe type IV developmental dysplasia. J Arthroplasty 32:1211–121920161115. 10.1016/j.arth.2016.11.005 27923597 10.1016/j.arth.2016.11.005

[5] Ors C, Caylak R, Togrul E (2022) Total hip arthroplasty with the Wagner cone femoral stem in patients with Crowe IV developmental dysplasia of the hip: a retrospective study. J Arthroplasty 37:103–109. 10.1016/j.arth.2021.09.007 34547428 10.1016/j.arth.2021.09.007

[6] Chen M, Gittings DJ, Yang S et al (2018) Total hip arthroplasty for Crowe type IV developmental dysplasia of the hip using a titanium mesh cup and subtrochanteric femoral osteotomy. Iowa Orthop J 38:191–195 30104944 PMC6047379

[7] Hua WB, Yang SH, Xu WH et al (2015) Total hip arthroplasty with subtrochanteric femoral shortening osteotomy for high hip dislocation. Orthop Surg 7:112–118. 10.1111/os.12176 26033991 10.1111/os.12176 PMC6583667

[8] Rogers BA, Garbedian S, Kuchinad RA et al (2012) Total hip arthroplasty for adult hip dysplasia. J Bone Joint Surg Am 94:1809–1821. 10.2106/JBJS.K.00779 23032592 10.2106/JBJS.K.00779

[9] Edwards BN, Tullos HS, Noble PC (1987) Contributory factors and etiology of sciatic nerve palsy in total hip arthroplasty. Clin Orthop Relat Res (218):136–141 3568473

[10] Farrell CM, Springer BD, Haidukewych GJ et al (2005) Motor nerve palsy following primary total hip arthroplasty. J Bone Joint Surg Am 87:2619–2625. 10.2106/JBJS.C.01564 16322610 10.2106/JBJS.C.01564

[11] Hasija R, Kelly JJ, Shah NV et al (2018) Nerve injuries associated with total hip arthroplasty. J Clin Orthop Trauma 9:81–8620171028. 10.1016/j.jcot.2017.10.011 29628688 10.1016/j.jcot.2017.10.011 PMC5884042

[12] Kong X, Chai W, Chen J et al (2019) Intraoperative monitoring of the femoral and sciatic nerves in total hip arthroplasty with high-riding developmental dysplasia. Bone Joint J 101–B:1438–1446. 10.1302/0301-620X.101B11.BJJ-2019-0341.R2 31674243 10.1302/0301-620X.101B11.BJJ-2019-0341.R2

[13] Vanlommel J, Sutter M, Leunig M (2020) Total hip arthroplasty using the direct anterior approach and intraoperative neurophysiological monitoring for Crowe III hip dysplasia: surgical technique and case series. Acta Orthop Belg 86:22–27 32490769

[14] Kabata T, Kajino Y, Inoue D et al (2019) Safety range for acute limb lengthening in primary total hip arthroplasty. Int Orthop 43:2047–2056. 10.1007/s00264-018-4158-6 30242514 10.1007/s00264-018-4158-6

[15] Reikeras O, Haaland JE, Lereim P (2010) Femoral shortening in total hip arthroplasty for high developmental dysplasia of the hip. Clin Orthop Relat Res 468:1949–1955. 10.1007/s11999-009-1218-7 20077043 10.1007/s11999-009-1218-7 PMC2881990

[16] Manafi Rasi A, Afzal S, Baroutkoub M et al (2025) Evaluation of active intraoperative nerve monitoring in severe developmental dysplasia of the hip patients undergoing total hip arthroplasty. Arthroplast Today 31:101612. 10.1016/j.artd.2024.101612 39898285 10.1016/j.artd.2024.101612 PMC11786204

[17] Bayram S, Akgül T, Özmen E et al (2020) Critical limit of Lower-Extremity lengthening in total hip arthroplasty: an intraoperative neuromonitorization study. J Bone Joint Surg Am 102:664–673 31977815 10.2106/JBJS.19.00988

[18] Murena L, Colin G, Dussi M et al (2021) Is intraoperative neuromonitoring effective in hip and pelvis orthopedic and trauma surgery? A systematic review. J Orthop Traumatol 22:40. 10.1186/s10195-021-00605-8 34647237 10.1186/s10195-021-00605-8 PMC8514601

[19] Taheriazam A, Baghbani S, Malakooti M et al (2024) Neuromonitoring in pre-post and intraoperative total hip replacement surgery in type 4 high-riding developmental dysplasia of the hip. Eur Rev Med Pharmacol Sci 28:98–106. 10.26355/eurrev_202401_34895 38235862 10.26355/eurrev_202401_34895

[20] Turan K, Kezer M, Çamurcu Y et al (2023) Intraoperative neurophysiological monitoring in total hip arthroplasty for Crowe types 3 and 4 hips. Clin Orthop Surg 15:711–717. 2023/10/09 37811513 10.4055/cios22371 PMC10551681

[21] Sutter M, Hersche O, Leunig M et al (2012) Use of multimodal intra-operative monitoring in averting nerve injury during complex hip surgery. J Bone Joint Surg Br 94:179–184. 10.1302/0301-620X.94B2.28019 22323682 10.1302/0301-620X.94B2.28019

[22] Bayram S, Akgul T, Ozmen E et al (2020) Critical limit of lower-extremity lengthening in total hip arthroplasty: an intraoperative neuromonitorization study. J Bone Joint Surg Am 102:664–673 2020/01/25. 10.2106/JBJS.19.00988 31977815 10.2106/JBJS.19.00988

[23] Hamrayev AJ, Buyukkuscu MO, Misir A et al (2020) The fate of femoral head autograft in acetabular reconstruction in dysplastic hips at midterm. J Orthop Surg (Hong Kong) 28:2309499020957109. 10.1177/2309499020957109 32996378 10.1177/2309499020957109

[24] Shi XT, Li CF, Han Y et al (2019) Total hip arthroplasty for Crowe type IV hip dysplasia: surgical techniques and postoperative complications. Orthop Surg 11:966–973. 10.1111/os.12576 31755242 10.1111/os.12576 PMC6904615

[25] Krych AJ, Howard JL, Trousdale RT et al (2009) Total hip arthroplasty with shortening subtrochanteric osteotomy in Crowe type-IV developmental dysplasia. J Bone Joint Surg Am 91:2213–2221 2009/09/03. 10.2106/JBJS.H.01024 19723999 10.2106/JBJS.H.01024

[26] Sloan TB, Heyer EJ (2002) Anesthesia for intraoperative neurophysiologic monitoring of the spinal cord. J Clin Neurophysiol 19:430–443. 10.1097/00004691-200210000-00006 12477988 10.1097/00004691-200210000-00006

[27] Langeloo DD, Journee HL, de Kleuver M et al (2007) Criteria for transcranial electrical motor evoked potential monitoring during spinal deformity surgery A review and discussion of the literature. Neurophysiol Clin 37:431–439. 10.1016/j.neucli.2007.07.007 18083499 10.1016/j.neucli.2007.07.007

[28] MacDonald DB, Dong C, Quatrale R et al (2019) Recommendations of the international society of intraoperative neurophysiology for intraoperative somatosensory evoked potentials. Clin Neurophysiol 130(20181114):161–179. 10.1016/j.clinph.2018.10.008 30470625 10.1016/j.clinph.2018.10.008

[29] Li H, Yuan Y, Xu J et al (2018) Direct leverage for reducing the femoral head in total hip arthroplasty without femoral shortening osteotomy for Crowe type 3 to 4 dysplasia of the hip. J Arthroplasty 33:794–799. 10.1016/j.arth.2017.09.011 29269273 10.1016/j.arth.2017.09.011

[30] Eggli S, Hankemayer S, Muller ME (1999) Nerve palsy after leg lengthening in total replacement arthroplasty for developmental dysplasia of the hip. J Bone Joint Surg Br 81:843–845. 10.1302/0301-620x.81b5.9610 10530847 10.1302/0301-620x.81b5.9610

[31] Deletis V, Sala F (2008) Intraoperative neurophysiological monitoring of the spinal cord during spinal cord and spine surgery: a review focus on the corticospinal tracts. Clin Neurophysiol 119:248–264. 10.1016/j.clinph.2007.09.135 18053764 10.1016/j.clinph.2007.09.135

[32] MacDonald DB (2017) Overview on criteria for MEP monitoring. J Clin Neurophysiol 34:4–11. 10.1097/WNP.0000000000000302 28045852 10.1097/WNP.0000000000000302

[33] Allison DW, Verma A, Holman PJ et al (2024) Transabdominal motor evoked potential neuromonitoring of lumbosacral spine surgery. Spine J 24:1660–167020240427. 10.1016/j.spinee.2024.04.029 38685276 10.1016/j.spinee.2024.04.029

[34] Yalinay Dikmen P, Ozden VE, Dikmen G et al (2019) Intraoperative neuromonitoring of anterior root muscle response during hip surgery under spinal anesthesia. J Clin Monit Comput 33(20181110):695–702. 10.1007/s10877-018-0212-6 30415323 10.1007/s10877-018-0212-6

